# The Human dsRNA binding protein PACT is unable to functionally substitute for the *Drosophila* dsRNA binding protein R2D2

**DOI:** 10.12688/f1000research.2-220.v2

**Published:** 2014-03-28

**Authors:** Benjamin K Dickerman, Jocelyn A McDonald, Ganes C Sen

**Affiliations:** 1 Department of Molecular Genetics, Lerner Research Institute, Cleveland Clinic, Cleveland, OH, 44195, USA

## Abstract

The dsRNA binding protein (dsRBP) PACT was first described as an activator of the dsRNA dependent protein kinase PKR in response to stress signals.  Additionally, it has been identified as a component of the small RNA processing pathway.  A role for PACT in this pathway represents an important interplay between two modes of post-transcriptional gene regulation.  The function of PACT in this context is poorly understood.  Thus, additional approaches are required to clarify the mechanism by which PACT functions.  In this study, the genetic utility of *Drosophila melanogaster* was employed to identify dsRNA-binding proteins that are functionally orthologous to PACT.  Transgenic *Drosophila* expressing human PACT were generated to determine whether PACT is capable of functionally substituting for the *Drosophila* dsRBP R2D2, which has a well-defined role in small RNA biogenesis.  Results presented here indicate that PACT is unable to substitute for R2D2 at the whole organism level.

## Introduction

The dsRNA binding protein (dsRBP) PACT (referred to as RAX in the mouse) activates the dsRNA dependent protein kinase PKR in response to various stress signals ^1– 5^. This in turn results in repression of translation through phosphorylation of the eukaryotic translation initiation factor eIF2α ^6^. Although the biochemical function of PACT as an activator of PKR is well established, the physiological role of this protein *in vivo* is unclear. Indeed, generation of mice lacking RAX revealed a role for this protein in craniofacial ^7, 8^ and anterior pituitary development ^9^ that is not readily explained by current knowledge of its biochemical function. More recently, PACT has been identified as a component of the RNA silencing (siRNA/miRNA) pathway ^10, 11^, although a detailed understanding of its role in this pathway is limited. Thus, PACT functions in two independent pathways that govern post-transcriptional gene regulation and as such it is important to understand the overall physiological function of this protein. To this end, we investigated the functional orthology between PACT and the *Drosophila melanogaster* dsRBP R2D2.

RNA silencing through the micro-RNA (miRNA) and short-interfering RNA (siRNA) pathways regulates nearly every aspect of cellular biology (reviewed by van Kouwenhove *et al.* ^12, 13^). Both siRNA and miRNA pathways require cytoplasmic processing of precursor molecules (either dsRNA or ~70bp pre-miRNAs) to generate mature guide RNAs (reviewed by van Kouwenhove *et al.* ^12^). In both cases, this cytoplasmic processing step is accomplished by the endonuclease Dicer in complex with one or more dsRNA binding proteins, which cleaves precursor RNA molecules into ~20–25bp dsRNA. One of the two strands of the Dicer-processed mature RNA molecule (known as the guide strand) is then loaded into the effector complex, the RNA-induced silencing complex (RISC) (reviewed by Czech and Hannon ^13^). Although the precise composition of the RISC is unclear, various studies have demonstrated the presence of a mature guide RNA, Dicer, one or more accessory dsRBPs, and one of the four members of the Argonaute protein family (Ago1–4) which function as the effector enzymes in the terminal silencing step ^14^.

The RNA silencing pathways of metazoans have largely been investigated in the model organisms *Caenorhabditis elegans* and *Drosophila melanogaster*. In both systems, it was discovered that the endonucleases required for cleavage of pri-miRNAs (Drosha) and pre-miRNAs/siRNA precursors (Dicer) form complexes with specific dsRBPs. These proteins, like PACT, are comprised of tandem dsRBDs with no other identifiable domains (Drosha complexes with Pasha/DGCR8 ^15^, *C. elegans* Dicer binds RDE-4 ^16^, *D. melanogaster* Dicer-1 binds Loquacious (LOQS) ^17^ and *D. melanogaster* Dicer-2 binds R2D2 ^18^). Furthermore, these dsRBPs are required for the processing functions of the complexes. In mammalian systems, processing of both siRNA and miRNA is performed by a common Dicer enzyme. Both PACT and a similar dsRBP Tat Trans-activation element RNA binding protein (TRBP) in human/Protamine-1 RNA binding protein (PRBP) in mouse have been demonstrated to bind Dicer through either their N-terminal dsRNA binding motifs or their C-terminal Merlin-Dicer-PACT Liaison (Medipal) domain (which contains the PKR activation domain of PACT) ^10, 11, 19^. The binding of PACT, TRBP or both with Dicer enhances the ability of Dicer to process dsRNA into siRNAs *in vitro* ^10^. These findings suggest a functional significance to the PACT/TRBP/Dicer interactions, although the mechanism by which this occurs is unclear. These reports suggest that in addition to the regulating PKR activation, PACT may affect post-transcriptional control via small RNA pathways.

**Figure 1.**
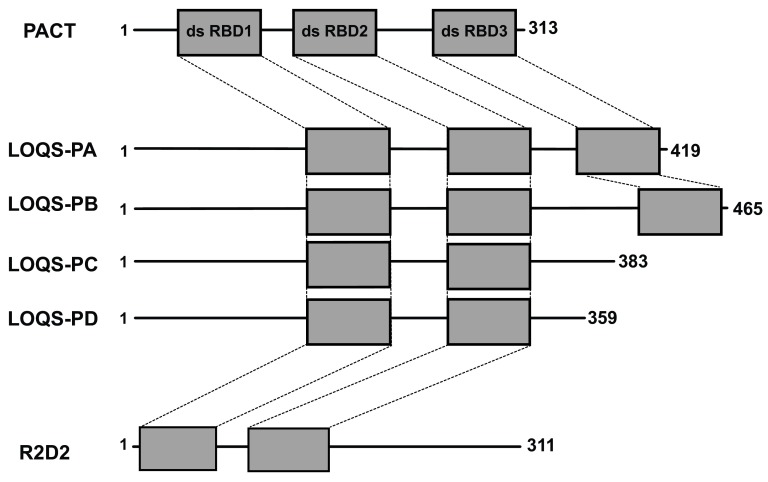
Schematic representation of PACT and Drosophila dsRBPs. Schematic diagram of the PACT, LOQS and R2D2 proteins depicting the organization of their dsRBD domains. Dashed lines designate dsRBD1, 2 or 3.

In *Drosophila*, processing of small RNA precursors is accomplished by separate enzymes, Dicer-1 for miRNA and Dicer-2 for siRNA ^20^. The dsRNA binding proteins LOQS ^17^ and R2D2 ^18^ bind to Dicer-1 and Dicer-2, respectively. LOQS and R2D2 sort precursor RNA molecules based on the presence or absence of mismatches in the stemloop of the hairpin or dsRNA precursor to the appropriate Dicer processing pathway ^23^ and facilitate RISC loading ^24, 25^. LOQS encodes four isoforms; LOQS-PA and LOQS-PB contain three tandem dsRBDs whereas both LOQS-PC and LOQS-PD ^21, 22^ lack the third C-terminal dsRBD. R2D2 encodes a single isoform that is comprised of two dsRBDs ^18^ ( Figure 1). Within the dsRBDs of these proteins, there is a high degree of sequence similarity (71–78% similarity, see Table 1) between PACT and LOQS PA and PB (which have all three dsRBDs in common). This sequence similarity coupled with the tandem dsRBD architecture (see Figure 1 and Table 1) indicates potential functional orthology between PACT and LOQS. Although R2D2 is significantly less similar to PACT (46% sequence similarity within dsRBD2, with no detectable similarity within dsRBD1), it has nonetheless been demonstrated to bind Dicer-2 ^18^ and possesses a tandem dsRBD architecture similar to PACT and LOQS and thus may also function orthologously to PACT/RAX.

**Table 1.** Amino acid sequence similarity between PACT and *Drosophila* dsRBPs. Amino acid sequence identity and similarity (positives) between dsRNA binding domains of PACT and the *Drosophila* dsRNA binding proteins determined by BLAST (blastp) alignment; ~ indicates no detectable similarity. The three dsRBDs shown for LOQS correspond to the shared dsRBDs of PA and PB isoforms; the PC and PD isoforms share the first and second but lack the third dsRBD.

		PACT dsRBD1		PACT dsRBD2		PACT dsRBD3	
		Identity	Similarity	Identity	Similarity	Identity	Similarity
LOQS	dsRBD1 dsRBD2 dsRBD3	55% ~ ~	71% ~ ~	~ 62% ~	~ 78% ~	~ ~ 57%	~ ~ 76%
R2D2	dsRBD1 dsRBD2	~ ~	~ ~	~ 26%	~ 46%	~ ~	~ ~

Previous studies have produced mutant *D. melanogaster* lines lacking either of the dsRBPs that interact with Dicer-1 and Dicer-2. Flies deficient in all four isoforms of LOQS display developmental lethality, and do not survive past the pupal stage ^26^. In contrast, flies homozygous mutant for a null allele of *r2d2* are semi-lethal but the surviving flies have severe fertility defects ^27^. The fertility phenotype results from defective oogenesis. Specifically, loss of R2D2 disrupts formation of the stalk cells, which normally separate individual follicles from each other ^28^. This phenotype is enhanced by a *dicer-1* mutant, indicating an unexpected interaction between R2D2 and Dicer-1 rather than Dicer-2 ^27^.

We hypothesized that one or more dsRNA binding proteins with known function in *D. melanogaster* are functionally orthologous to PACT, and that identification of the functional ortholog(s) would provide insight into the small RNA processing function of PACT. We sought to identify proteins orthologous to PACT by transgenically expressing human PACT in the well-characterized *Drosophila* genetic model system. We report here our efforts to determine the degree to which human PACT is a functional ortholog of *Drosophila* R2D2.

## Materials and methods

### Cell lines, plasmids and antibodies

HeLa cells (ATCC CCL-2) were maintained in Dulbecco's modified Eagle's medium (DMEM) containing glucose (4.5g/L), penicillin (50U/ml), streptomycin (50µg/ml), L-glutamine (2mM) and sodium pyruvate (1mM) (Cleveland Clinic Lerner Research Institute Media Preparation Core) supplemented with 10% fetal bovine serum (Atlanta Biologicals) at 37°C in 5% CO _2_. The pUASP vector was obtained from Drosophila Genomics Resource Center. A polyclonal rabbit antibody raised against full length PACT (produced in-house) ^29^ was used at 1:8000 and was detected using 1:10000 horseradish peroxidase (HRP) conjugated polyclonal goat anti-rabbit secondary (Rockland Immunochemicals # 611-103-122).

### Generation of pUASP plasmids

To obtain PACT cDNA, total RNA was extracted from HeLa cells using Trizol (Invitrogen) according to the manufacturer’s instructions, followed by DNAse treatment using the DNA-free kit (Ambion). Extracted, DNase-treated RNA was then reverse transcribed with the Superscript III system (Invitrogen) using random hexamers. The resulting cDNA was used to amplify the coding sequence of PACT by PCR using the primers 5´BamHI-PACT(UAS) (AAG GAT CCA AAC ATG TCC CAG AGC AGG CAC C) and 3´XbaI-PACT (GGC GGA TCC TTA CTT TCT TTC TGC TAT TAT CTT TAA ATA C). The resulting product was digested with BamHI (New England Biolabs Inc. #R0136) and XbaI (New England Biolabs Inc. #R0145) and ligated into pUASP to generate pUASP-PACT. The full-length *Drosophila r2d2* cDNA was amplified by PCR from a plasmid obtained from Drosophila Genome Research Center (cDNA #LD06392) using 5´BamHI-R2D2(UAS) (AAG GAT CCA AAC ATG GAT AAC AAG TCA GCC GTA TC) and 3´XbaI-R2D2 (AAA TCT AGA TTA AAT CAA CAT GGT GCG AAA ATA GTC TAT TAT ATG G). The resulting product was digested with BamHI and XbaI and ligated into pUASP to generate pUASP-R2D2. Final cloned plasmids were sequence verified using the primers listed above by the Cleveland Clinic Lerner Research Institute Genomics Core.

### Protein isolation and western blot

Protein was isolated by homogenizing individual adult flies in Triton X-100 lysis buffer (20mM Tris-HCl pH 7.5, 150mM NaCl, 1% Triton X-100, 1mM EDTA, 5mM 2-mercaptoethanol, 10% glycerol, supplemented with Complete protease inhibitor and PhoSTOP (Roche)). Protein was separated by sodium dodecylsulfate polyacrylamide gel electrophoresis (SDS-PAGE) and transferred to polyvinylidene fluoride (PVDF) membrane (Immobilon, Millpore) for western blot analysis. Western blots were visualized using enhanced chemiluminescence (ECL) detection reagents (GE Healthcare Lifesciences #RPN2106).

### *Drosophila* strains and genetics

Flies were maintained and crossed at 25°C according to standard protocols ^30^. Fly food was prepared according to the standard recipe from Bloomington Drosophila Stock Center with minor modifications (83.3ml/L molasses, 33.75g/L yeast, 97.58g/L cornmeal, 31g/L agar, 15.67ml/L Tegosept, all purchased from Genesee Scientific). Transgenic UAS- *PACT* and UAS- *r2d2* flies were generated ^31^ by Model Systems Genomics (Duke University, Durham, NC) using the plasmids pUASP-PACT and pUASP-R2D2 described above. The following fly strains were obtained from the Bloomington Drosophila Stock Center: *w ^1118^*, *r2d2 ^1^*/CyO (a null allele; ^27^) and Df(2L)BSC142/CyO. Tubulin ( *tub*)-GAL4 (insert on X) was a gift of Dr. A. Page-McCaw. Homozygous viable *r2d2 ^1^* flies were only obtained infrequently, suggesting that the stock acquired a second-site lethal mutation. Therefore, all experiments were performed by outcrossing *r2d2 ^1^* to the deficiency Df(2L)BSC142 (deletion of 28C3; 28D3, including the *r2d2* gene at 28C4). Standard genetic methods were used to construct the following genotypes: ^1^ *tub*-Gal4; *r2d2 ^1^*/CyO; ^2^ Df(2L)BSC142/CyO; UAS- *PACT*; and ^3^ Df(2L)BSC142/CyO; UAS- *r2d2.*

### Egg collection and hatch rate determination

Females of the genotype *tub*-GAL4; *r2d2 ^1^*/Df(2L)BSC142; +/UAS-x (where x is *PACT* or *r2d2*) were outcrossed with *w ^1118^* males for fertility tests. Crosses to test fertility were established in vials of food for 24 hours prior to transferring flies to an egg collection chamber. Females were allowed to lay eggs on grape juice agar plates (Genesee Scientific, #47–102) for 1 hr prior to collecting the plate, and incubating at 25°C for 24 hours to allow eggs to hatch. The total number of eggs laid was determined by counting the number of larvae and the number of unhatched eggs. Hatch rate was calculated as the number of larvae divided by the number of total eggs laid. Student’s t-test of two independent experiments (defining P < 0.05 as statistically significant) was performed using Graphpad Prism version 6.02 software package (Graphpad Software).

## Results

### Generation of human PACT-expressing transgenic *Drosophila melanogaster*

We reasoned that the genetically tractable *Drosophila* model system could be used to study the *in vivo* functions of PACT. Because there is no known PKR ortholog in *Drosophila*, this also allows the role of PACT in small RNA processing to be investigated independent of PKR activation. Moreover, the well-characterized siRNA and miRNA processing pathways and availability of *loqs* and *r2d2* mutant alleles make this an attractive system to study the role of PACT in the small RNA pathways. Various human proteins have been used to rescue *Drosophila* mutant phenotypes in order to identify homologous and orthologous proteins ^32– 34^, therefore to identify proteins orthologous to PACT, a human PACT transgene was constructed and introduced into *Drosophila*. This transgenic line was used to determine whether PACT can substitute for *Drosophila* dsRNA binding proteins that regulate small RNA processing (see below).

Human PACT-expressing *D. melanogaster* were created using the Gal4-UAS bipartite expression system ^35^. This system allows the generation of flies containing transgenes of interest under the transcriptional control of the UAS element. These lines then can be crossed to a number of Gal4-driver flies to tailor transgene expression to the needs of a specific experiment. The full-length coding region of the Human *PACT* gene was cloned into the pUASP vector under the transcriptional control of the UASp element, a modified form of UAS that permits expression in both somatic and germline cells ^36^. Several UAS- *PACT* transformants were obtained and their chromosomal insertions mapped. A stock that carried an insert on chromosome 3 was retained and crossed to the ubiquitous *tub*-GAL4 driver.

**Figure 2.**
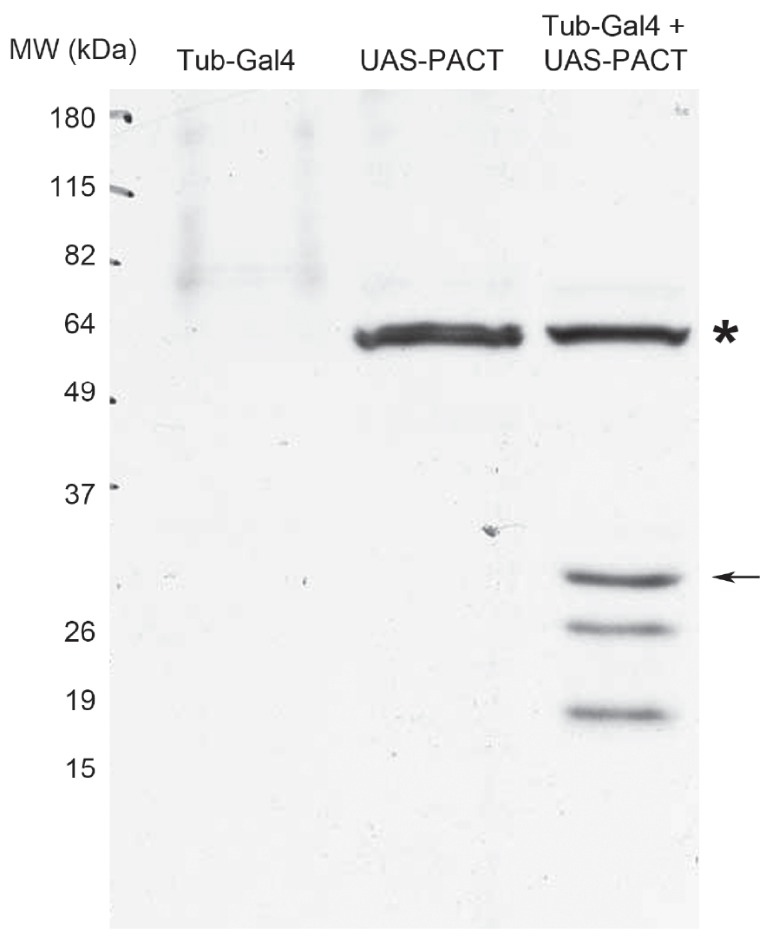
Expression of human PACT in transgenic Drosophila. Western blot analysis of total protein isolated from whole flies of the indicated genotypes. Flies carrying both the *tub*-Gal4 and UAS- *PACT* elements show a protein band of expected size for PACT that is not present in either stock alone; a non-specific band is indicated (*). Arrow denotes predicted molecular weight of Human PACT (34 kDa). Additional bands of smaller size likely represent degradation products.

We next tested whether Human PACT was expressed at detectable levels *in vivo*. Flies expressing *tub*-Gal4 alone, containing the UAS- *PACT* transgene alone, and flies with *tub*-Gal4 driving UAS- *PACT* were analyzed by western blot for PACT expression in whole flies. Using an antibody that recognizes Human PACT ^29^, we detected a protein band of the expected size (~34 kDa) in *tub*-GAL4; UAS- *PACT* flies but not in the individual parental lines ( Figure 2). Importantly, because we obtained viable *tub*-GAL4; UAS- *PACT* adult flies, this experiment also demonstrates that ubiquitous transgenic expression of PACT does not impair *Drosophila* development.

### Expression of human PACT is insufficient to overcome the *Drosophila* R2D2 deficient phenotype

The focus of the work presented here is on PACT rescue of the *r2d2* loss-of-function mutant phenotype. Investigation of the functional substitution between human PACT and *Drosophila* LOQS was investigated in a separate project in our lab and will be reported elsewhere.

**Figure 3.**
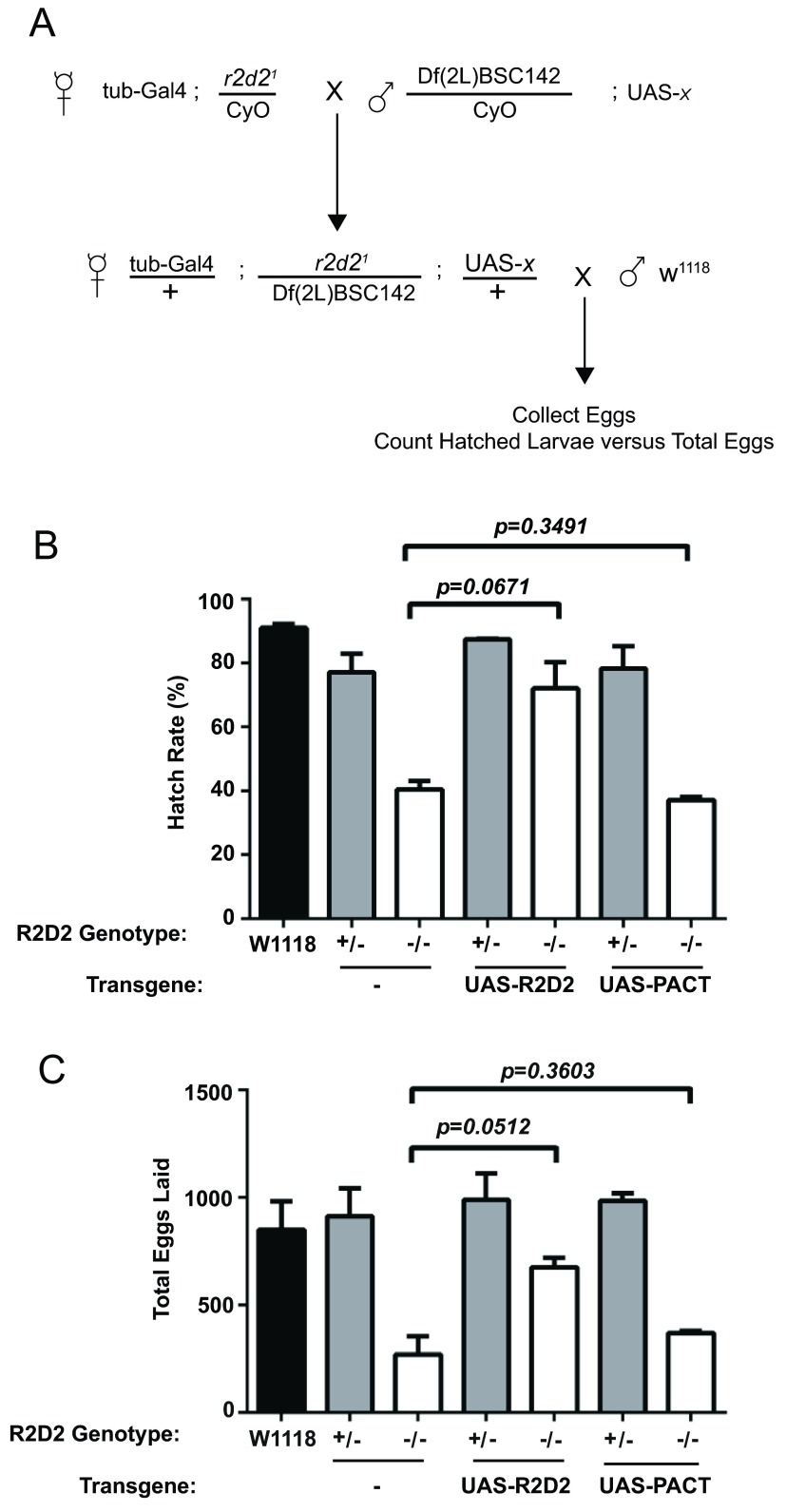
Rescue of the R2D2 deficient fertility defect by transgenic expression of R2D2 but not human PACT. ( **A**) Schematic representation of the experimental cross used to test fertility. UAS-x refers to the transgene shown in ( **B**) and ( **C**), for control crosses with no transgene the genotype Df(2L)BSC142/CyO; UAS-x was replaced with DF(2L)BSC142/CyO. ( **B**) Calculated hatch rate of eggs produced by wild-type ( *w ^1118^*) flies, flies with one copy of *r2d2* (+/-; either *r2d2 ^1^*/CyO or Df(2L)BSC142/CyO) and flies lacking both copies of *r2d2* (-/-, *r2d2 ^1^*/Df(2L)BSC142) and either expressing no UAS-transgene or expressing UAS- *PACT* or UAS- *r2d2*; these flies were outcrossed to *w ^1118^* males for the fertility tests. ( **C**) Total number of eggs laid from the crosses depicted in ( **B**). Graphs show mean ± SEM of two independent experiments, p-values were calculated by Student’s t-test.

Flies mutant for *r2d2* have reduced fertility ^27^. Therefore, we sought to determine the extent to which PACT can suppress *r2d2* mutant fertility defects. We performed fertility tests using a standard egg-laying and hatching assay. Crosses were performed to obtain female progeny that carried the *r2d2 ^1^* allele over a deficiency that removes the *r2d2* genetic locus (i.e. *r2d2*–/–), and simultaneously carried *tub*-GAL4 driving either UAS- *PACT* or UAS- *r2d2* ( Figure 3A; see Materials and methods for details). These female F1 progeny were then outcrossed to *w ^1118^* males to test for fertility; a cross of *w ^1118^* females to *w ^1118^* males was used as the wild-type control. Eggs resulting from these outcrosses were collected on grape juice agar plates, and allowed to hatch for 24 hours at 25°C. The total number of eggs produced and the number of hatched larvae were counted for each cross and hatch rate was calculated as the number of hatched larvae divided by the total number of eggs ( Figure 3B & 3C). Flies heterozygous for *r2d2* (either *r2d2 ^1^*/+ or Df(2L)BSC142/+) produced eggs and hatch rates equivalent to the wild-type control. Notably, expression of either UAS- *PACT* or UAS- *r2d2* in *r2d2* heterozygous flies did not influence fertility or hatch rates. Consistent with a previous report ^27^, flies homozygous mutant for *r2d2* had a strong reduction in both the total number of eggs laid and the hatch rate compared to wild-type ( Figure 3). We observed a rescue of both total number of eggs (p=0.0512) and hatch rate (p=0.0671) in *r2d2* mutant flies by ubiquitous ( *tub*-GAL4) transgenic expression of UAS- *r2d2* that is approaching statistical significance and consistent with previous reports ^27^. In contrast, ubiquitous expression of UAS- *PACT* did not suppress the homozygous *r2d2* mutant fertility defects; the hatch rate and number of eggs laid was equivalent to the *r2d2* mutants alone ( Figure 3). Despite a documented interaction between PACT and the mammalian small RNA processing machinery and similar domain architecture between these proteins, these results demonstrate that Human PACT is not sufficient to rescue the R2D2 deficient phenotype.

## Discussion

Identification of PACT as a component of the small RNA processing pathway implicates this protein in multiple pathways of post-transcriptional gene regulation through general inhibition of translation by PKR and target-specific regulation by siRNA/miRNA. As such it is important to determine the precise nature of PACT’s involvement in this pathway in order to understand the overall function of PACT in post-transcriptional gene regulation, and the physiological effects of the protein. Here, we investigated the role of PACT in RNA silencing by attempting to identify orthologous proteins in *D. melanogaster*. Human PACT was introduced into *Drosophila* to determine whether it could substitute for R2D2, which has a well characterized function in RNA silencing. Expression of PACT was unable to rescue the phenotypes of flies lacking R2D2. These results suggest that the Dicer-associated dsRBPs PACT and R2D2 are not functionally orthologous.

While our study demonstrates that Human PACT cannot substitute for R2D2 function in flies, there are a number of important caveats to the results described here. Despite the lack of rescue, it is nonetheless possible that PACT plays the same (or a similar) role in mammalian cells as R2D2 plays in *Drosophila* cells. One possibility could be that PACT protein is sufficiently different from R2D2 that it cannot interact *in vivo* with *Drosophila* partner proteins that are necessary for R2D2 function. Alternatively, R2D2 may be required to bind specific RNA precursor molecules that PACT is unable to bind (or has a lower affinity for). Investigation of biochemical interactions between PACT and *Drosophila* RNA silencing proteins such as Dicer-1 and Dicer-2 and a biochemical comparison of the specific RNA binding functions of these proteins would begin to address these issues.

In addition to functional discrepancies between these proteins, there may be other potential explanations for these results that would not rule out orthologous function between PACT and R2D2. Although less likely, the lack of PACT rescue of the *r2d2* mutant may be due to inherent difficulty in controlling the tissue-specificity and relative levels of transgene expression *in vivo*. In the case of R2D2, expression in the stalk and follicle cells of the ovary is required for egg development ^27^. Expression of UAS- *PACT* by *tub*-GAL4 in whole flies was validated by western blot; however, this does not provide information about transgenic expression of PACT in the appropriate tissues. Thus, even though PACT expression was driven by a ubiquitous GAL4 driver we cannot rule out insufficiently high expression of PACT in the relevant cell types. Expression of UAS- *R2D2* using the same *tub*-GAL4 driver however rescued the *r2d2* loss-of-function fertility defect. Although this result provides some degree of confidence in the expression levels, the genomic context of the P-element insertion and stability of transgenically-produced RNA and protein can all contribute to expression variability *in vivo*. As such, it still remains a possibility that PACT was not expressed as highly as R2D2 using the same transgenic expression system, and thus PACT may not have rescued fertility due to insufficient expression rather than a lack of orthologous function. This issue could be addressed in future experiments by expressing epitope-tagged R2D2 or PACT in *r2d2* mutant flies to correlate rescue of the *r2d2* deficient phenotype with a direct comparison of protein expression level ^37^. Additional experiments investigating tissue-specific expression levels of these proteins as well as using tissue-specific GAL4 drivers would begin to address these concerns.

While the complementation experiments described here indicate that PACT is unable to functionally substitute for R2D2, there are complications to the interpretation of these experiments. Further technical refinements will be necessary to definitively demonstrate or rule out functional orthology between PACT and R2D2. It also remains a distinct possibility that PACT is instead orthologous to LOQS, a topic that we are interested in investigating further in the future.

## Figures and Tables

**Table S1.** Hatch rate and egg count raw data replicate 1.

	Wild type	No transgene		UAS-R2D2		UAS-PACT	
	W1118	+/-	-/-	+/-	-/-	+/-	-/-
Larvae	884	743	153	969	403	728	145
Eggs laid	983	1042	355	1112	631	1021	380
% Hatched	89.92879	71.30518	43.09859	87.14029	63.86688	71.30264	38.15789

**Table S2.** Hatch rate and egg count raw data replicate 2.

	Wild type	No transgene		UAS-R2D2		UAS-PACT	
	W1118	+/-	-/-	+/-	-/-	+/-	-/-
Larvae	663	651	71	763	579	809	131
Eggs laid	718	785	187	870	721	948	361
% Hatched	92.33983	82.92994	37.96791	87.70115	80.30513	85.33755	36.28809
