# Supplementary material for: The Human dsRNA binding protein PACT is unable to functionally substitute for the Drosophila dsRNA binding protein R2D2
Source: F1000Res. 2014 Mar 28;2:220. Originally published 2013 Oct 18. [Version 2] doi: 10.12688/f1000research.2-220.v2 (PMC3962003; doi:10.12688/f1000research.2-220.v2)
Supplement: Peer review history

# Peer review: Referee response for version 2

**DOI:** 10.5256/f1000research.4070.r4717

**Published:** 2014-05-09

**Authors:** Anthony Sadler

**Affiliations:** 1 Monash Institute for Medical Research, Monash University, Melbourne, Australia

I have read this submission. I believe that I have an appropriate level of expertise to confirm that it is of an acceptable scientific standard.

# Peer review: Referee response for version 1

**DOI:** 10.5256/f1000research.2593.r2387

**Published:** 2014-02-18

**Authors:** Anita Corbett

**Affiliations:** 1 Emory University, Atlanta, GA, USA

The manuscript by Dickerman *et al.* describes an attempt to study the functional conservation of RNA binding proteins implicated in processing of small RNAs and activation of dsRNA-dependent protein kinase (PKR).  The work exploits a Drosophila model where PKR is not relevant thus allowing focus on the small RNA processing pathways. The approach is logical to try to rescue a fertility phenotype observed for the Drosophila protein (R2D2) with transgenic expression of a related human protein, PACT.  Unfortunately, the authors were not able to demonstrate rescue and thus are left with a negative result.

The data presented do support the conclusions drawn, that no rescue could be detected. There are a number of caveats for this result as the authors describe in the Discussion.

This is a reasonable contribution to the literature in that it would prevent other researchers from wasting time undertaking the identical experiment. The data presented is robust and convincing.

I would suggest some modifications to the text for clarification:

 

1. The authors begin by describing both related human proteins, PACT and the isoforms of LOQS.  While it is logical to describe the family of proteins, this study actually only analyzes the PACT protein. This point should be clearly communicated earlier in the Introduction to the study not just as the final sentence of the Introduction.
2. The section in the Introduction describing Figure 1 is very convoluted and really could be re-written to be clearer. An example with grammatical errors is the second sentence of this paragraph, which reads: *“The dsRNA binding proteins LOQS and R2D2 bind Dicer-1 to Dicer-2 respectively.” *I think what the authors mean to say is: *“The dsRNA binding proteins, LOQS and R2D2, bind TO Dicer-1 AND Dicer-2, respectively.” *
  The description of the proteins really needs to be rewritten for clarification.
3. If the authors could perform an additional experiment to address one of the caveats that they mention, ideally they could compare the expression levels for the transgenic human and fly proteins. Such a direct comparison would really only be possible if the proteins are tagged with an epitope tag.

 

Given the number of caveats for the study presented here, the real value as described above is simply to communicate that these experiments have been attempted using this approach.

I have read this submission. I believe that I have an appropriate level of expertise to confirm that it is of an acceptable scientific standard.

# Response

**Published:** 2014-04-03

**Authors:** Benjamin Dickerman

**Affiliations:** University of Melbourne, Australia

We have addressed the comments presented in a revised manuscript. A sentence reading “ *To this end, we investigated the functional orthology between PACT and the Drosophila melanogaster dsRBP R2D2.*” has been added to the end of the first paragraph of the introduction, to highlight the focus of this manuscript before describing the family of dsRBPs. The paragraph describing figure 1 has been reordered and edited for clarity, and to address the specific grammatical error mentioned by the reviewer. A sentence reading “ *This issue could be addressed in future experiments by expressing epitope-tagged R2D2 or PACT in r2d2 mutant flies to correlate rescue of the r2d2 deficient phenotype with a direct comparison of protein expression level.*” has been added to the relevant paragraph of the discussion section suggesting this experiment as part of future studies.  This has been referenced as a comment by the reviewer to credit the suggestion.

# Peer review: Referee response for version 1

**DOI:** 10.5256/f1000research.2593.r2676

**Published:** 2013-12-06

**Authors:** Anthony Sadler

**Affiliations:** 1 Monash Institute for Medical Research, Monash University, Melbourne, Australia

This is a useful study that is well executed and that makes a valuable contribution. The results are clearly described. An attempt to address the acknowledged uncertainties in this study, by measuring the levels of PACT in the critical tissues, comparison of the levels of expression of PACT to the transgenic R2D2, detecting an association between human PACT and Drosophila Dicer-2, and/or otherwise confirming function of the PACT transgene would have increased confidence in the conclusion. These measures would be less germane if the parallel experiment to rescue the function of Drosophila LOQS, which is the more likely homolog for PACT, had demonstrated the functionality of the PACT transgene. Perhaps a sentence could be introduced into the discussion that these separate experiments validated the PACT transgene functioned in Drosophila - if this was the case.

I have a number of very minor criticisms that might improve the manuscript.

The statement in the abstract that the primary function of PACT is to activate PKR is not accurate. As the authors themselves state the relative physiological consequence of PACT, in small RNA biogenesis or protein translation, is uncertain. This sentence might be better expressed by, for instance, writing PACT was first described to function by activating PKR.

The sentence in the abstract stating that additional models are required to clarify the function of PACT does not best justify the study approach. Alternatively, describing the utility of Drosophila to decipher small RNA biogenesis would seem more appropriate.

It might improve the clarity of the text if the authors selected a simpler abbreviation, rather than PACT/RAX, such as either PACT or RAX.

I have read this submission. I believe that I have an appropriate level of expertise to confirm that it is of an acceptable scientific standard.

# Response

**Published:** 2014-04-03

**Authors:** Benjamin Dickerman

**Affiliations:** University of Melbourne, Australia

We agree that inclusion of LOQS rescue data would strengthen the arguments presented in this manuscript.  Despite repeated efforts, and likely due to technical limitations, *Drosophila* LOQS (UAS-LOQS-PB) was unable to rescue *loqs* loss-of-function mutations in our hands. This important control has been previously reported ( Park *et al.,* 2007) to be sufficient to rescue the *loqs* loss-of-function mutation that was used in this study.  Because these rescue studies were inconclusive, we are currently unable to confirm that LOQS and PACT are functional orthologous.

We have addressed the remaining comments presented here in a revised manuscript. The abstract has been revised to clarify stress-induced PKR activation as the first described rather than the primary function of PACT, as well as to highlight the genetic utility of Drosophila as a model system, rather than merely suggesting that additional model systems are required. To address the final comment regarding PACT nomenclature, the manuscript has been revised for clarity by changing PACT/RAX to PACT unless specifically referring to the mouse protein RAX.
